# Religiosity, Theism, Perceived Social Support, Resilience, and Well-Being of University Undergraduate Students in Singapore during the COVID-19 Pandemic

**DOI:** 10.3390/ijerph20043620

**Published:** 2023-02-17

**Authors:** Samuel Ken-En Gan, Sibyl Weang-Yi Wong, Peng-De Jiao

**Affiliations:** 1 Wenzhou Municipal Key Lab of Applied Biomedical and Biopharmaceutical Informatics, Wenzhou-Kean University, Wenzhou 325015, China; 2 Department of Psychology, James Cook University, Singapore 387380, Singapore; 3 Antibody & Product Development Lab, APD SKEG Pte Ltd., Singapore 439444, Singapore; 4 Zhejiang Bioinformatics International Science and Technology Cooperation Center, Wenzhou-Kean University, Wenzhou 325015, China

**Keywords:** religion, religiosity, well-being, resilience, perceived social support, COVID-19 pandemic, beliefs, theism

## Abstract

The COVID-19 pandemic infection control measures severely impacted mental well-being, allowing insight into possible protective parameters. With religion playing a role during challenging times, this study investigated theism and religiosity on the mental well-being of university students during the COVID19 pandemic and how social support and resilience can mediate this effect. One hundred eighty-five university students between 17 and 42 years old responded to online surveys on their theism, religious affiliations, religiosity, well-being, perceived support, and resilience. Pearson’s correlations and single and sequential mediation analyses showed that theism did not significantly predict well-being (r = 0.049), but religiosity mediated the relationship (r = 0.432, effect size = 0.187). Sequential mediation analysis showed that resilience did not mediate the relationship between religiosity and well-being, but perceived social support significantly positively mediated religiosity and well-being with an effect size of 0.079. The findings reveal that factors, such as religiosity and social support could thus aid in the mental well-being of future challenging times such as the pandemic.

## 1. Introduction

The global COVID-19 pandemic measures augmented isolation, loss of jobs, and fear of death and illnesses [1] to elicit increased stress and anxiety, including those of underlying financial reasons to adversely affect the mental health and well-being of individuals [2]. University students are one particular susceptible group to mental health issues [3], yet the effects of the COVID-19 pandemic on students are yet to be extensively studied. For this group, the pandemic control measures negatively affected future career opportunities through the disruption of learning [4] amongst the acquisition of skills, thus requiring substitution teaching methods such as video demonstrations instead of live demonstrations [5] that increased the burden in learning with additional manipulation of digital tools. 

In such times of crises, many people instinctively seek for underlying reasons to justify their vulnerabilities [6], and religion has long been used to explain crisis-induced vulnerabilities [7]. Some individuals attributed the COVID-19 pandemic to the work of supernatural beings [8], and an increase in prayer duration was observed for 61% of Poles [9], while ~30% of Americans reported strengthened faith [10] during the pandemic. There is thus an association between organized religion and well-being [11], where organized religion can provide comfort via extensive and integrated reasoning structures to justify challenging life circumstances [12] and likely peer support. Albeit, contradictory findings were also reported with different groups of people [13,14].

Nonetheless, religiosity and psychological distress were found to be synergistic [15,16,17] with social support [18] to increase resilience [13,14] against psychological distress. A positive mindset and functioning [19,20] was found contributed by religious affiliations [21,22,23,24] to provide a sense of hope in tumultuous times to preserve control, sense of meaning, and esteem [6,25,26,27,28]. Tan et al. [29] found a positive correlation between religion and mental health among older Malaysian adults, while Aydogdu et al. [30] found religiosity to positively correlate with higher levels of happiness and life satisfaction. However, Murken [31] found no direct association between religiosity and well-being, suggesting that the effect may be significant only in highly religious individuals [31].

Investigating the impact of religion and social support on happiness, Formoso-Suárez et al. [32] found a positive correlation between happiness and satisfaction with the absence of negative emotions, leading to better mental health. Support and positive emotions were later also linked by Formoso-Suárez, Saiz, Chopra, and Mills [32] to support the broaden-and-build theory of positive emotions, where positive emotions built various personal resources such as physical resources (e.g., health), social resources (e.g., social support), and psychological resources (e.g., resilience) [33]. Reviewing psychological interventions for adult resilience enhancement, Helm Helmreich [34] found associations between resilience and several factors: self-efficacy, coping ability, social support, religious and spiritual beliefs, positive emotions, self-esteem, and meaning in life.

As the ‘support accessible to an individual through social ties to other individuals, groups, and the larger community’ [35,36], social support is critical to coping with stressful events and is essential in mediating the pressures of stress [37]. Perceived social support could predict mental health better than many other measures [38] and is frequently measured [39]. It can come from family, friends, romantic partners, pets, community, and colleagues [36] as well as from religious group activities to provide comfort against stressors through tangible and emotional help [40,41]. In fact, patients with perceived low social support reported significantly higher levels of depression and it is negatively correlated with long-term psychological distress. Interestingly, the size of the support network can buffer against trait anxiety and depression [42]. Stronge, Overall, and Sibley [41] found a strong positive relationship between perceived social support and well-being (i.e., life satisfaction and self-esteem), further extending the connections.

Social support and resilience can protect mental health [43,44] and promote well-being [45]. Since resilience is the ability to ‘bounce back’ or recover from stress [46] to handle stressors more effectively [47], negative associations between resilience and stress [48,49] suggested that the former acted as long-lasting personal support [49], where highly resilient individuals tend to elicit more positive emotions and positive meaning in their everyday life stressors than low-resilient individuals [50]. As resilience was negatively correlated with poor mental health indicators (i.e., negative emotions and depression) [51,52] during the COVID-19, resilient individuals who were more optimistic, curious, open, relaxed, creative, and had more zeal [48,53,54,55] were able to better create positive emotionality to cope with adversities [48].

Exercise is reported to positively affect well-being [56,57], mitigate stress [58], and distract from stressors to pause daily activities [59]. Nonetheless, it has been reported to reduce anxiety [60] and improve well-being when performed regularly. It can elicit some of these positive effects through its association with lower hypothalamic–pituitary–adrenal (HPA) axis reactivity [61,62] for adaptive reactions to psychological stress [63] related to mental ill-being [64]. With the HPA axis being one of the neurotransmitters in resilience [65], exercise could therefore positively affect resilience [66,67], where exercise increased galanin levels, a neuropeptide of the locus coeruleus [68] to help stress resilience [69]. Since the infection control measures of the COVID-19 pandemic caused a change in exercise behaviors worldwide [57], exercise behaviors was also investigated as a confounding variable in this study.

During the pandemic, resilience and religiosity supported well-being [70,71] by providing intrinsic social support developed from religious beliefs that supplied a relationship with the Divine and meaning to distressing events [72]. Studying hemodialysis patients, Freire de Medeiros et al. [73] found a positive association between resilience and religiosity but negative correlations between resilience and depression and also between religiosity and depression. Similarly, Fradelos et al. [74] also found positive correlations between religiosity and resilience, but no associations were found between religiosity and mental health factors (i.e., anxiety and depression), while Mosqueiro et al. [75] found a positive relationship between religiosity, resilience, and higher quality of life and an inverse relationship between religiosity and suicide attempts. Altogether, these findings seem to agree that resiliency mediated the relationship between religiosity and mental well-being.

It was suggested that the contradicting findings on religion and well-being could be attributed to differences in the perceived closeness to the Divine [16], the lack of required minimum sample size [15], or the method used (e.g., Duke University Religion Index did not measure religious coping), together with the fact that most previous studies tend to focus only on religiosity [11,31], particularly extrinsic religiosity [76,77] and intrinsic religiosity [78], leaving much to investigate. However, it should be noted that religion can encompass many parameters that are not necessarily coupled tightly. For example, the belief in the supernatural (which can include ghosts, fairies, etc.) is distinct from theism (the belief in a higher being(s) [79]) and from religiosity (the engagement of organized systematic structure of beliefs and practices [27,28,80,81,82]). Considering that there can be people who are part of a religious group for social purposes without the prescribed theism and vice versa, there is a need to study theism and religiosity separately for a more in-depth investigation into the effects of religion on the whole. In fact, the positive association found between religiosity and social support where social support mediated the relationship between intrinsic religiosity and well-being (Milevsky [83]) supports the distinction of belief and practice, and may explain Revens, Gutierrez, Paul, Reynolds, Price, and DeHaven’s [14] proposal that social support mitigated psychological distress in the lack of direct effects of religiosity on psychological distress.

This study thus investigated whether the effects of theism and religiosity on the mental well-being of university students during the COVID19 pandemic were potentially protective, and if so, how social support and resilience can mediate this effect. Addressing all the parameters of beliefs (Hypothesis 1), religiosity (Hypothesis 2), resilience, social support, and exercise (latter 3 in Hypothesis 4) would be investigated with mental well-being in this study.

Specifically:

**Hypothesis** **1** **(H1).** *Theists would report higher well-being than atheists.*

**Hypothesis** **2** **(H2).** *Those with higher religiosity scores would have better well-being scores.*

**Hypothesis** **3** **(H3).** *Theists would report higher well-being due to higher religiosity.*

**Hypothesis** **4** **(H4).** *Higher religiosity scores would be associated with higher resilience and social support to give rise to better well-being.*

## 2. Materials and Methods

### 2.1. Design

This study utilized a cross-sectional design with two mediation analyses and only one dependent variable (DV), well-being, which was operationalized as mental well-being. The Statistical Package for Social Science (SPSS) software version 25 by IBM and PROCESS for SPSS Version 3.5.1 were used to conduct the analyses of this study.

### 2.2. Mediation Analysis 1

**Figure 1:**
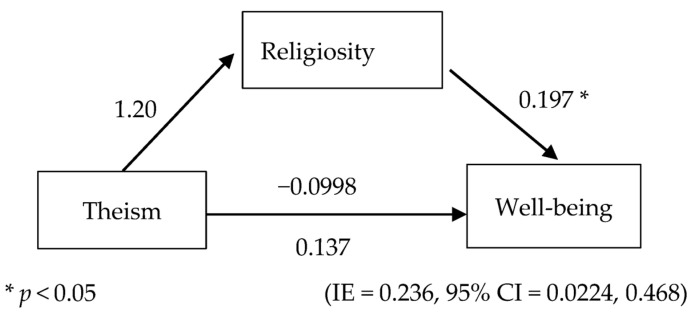
Statistical model of beliefs in God(s) as theism, religiosity, and well-being.

The predictor in this mediation analysis was theism: the belief in God(s) or belief in the absence of a God using the Paranormal Beliefs Questionnaire (Supplementary Materials). The independent variable (IV) in theism, mediator (religiosity), exercise, and dependent variable (DV) well-being were analyzed for correlations. Theism was recoded from the Paranormal Belief Questionnaire into 1 = Theists (i.e., ‘There is one God’, ‘There are multiple Gods’, ‘There might be a God, or Gods’, and ‘Everything is God’), and 0 = Atheists (i.e., ‘There is no God’). Theism was chosen because the common factor in the structural definition of any religion is the belief in God or the Divine, recognized as either an immanent or superior being [84,85], or lack thereof. Therefore, it was deemed a suitable classification for this analysis with the scores of the Interreligious Centrality of Religiosity Scale (CRSi-20). Theism was specifically used to differentiate from religious beliefs to take into account the likely dissonance present within various organized religions. The mediation analysis figure is shown in Figure 1. Exercise behavior was also studied as a covariate.

### 2.3. Mediation Analysis 2

**Figure 2:**
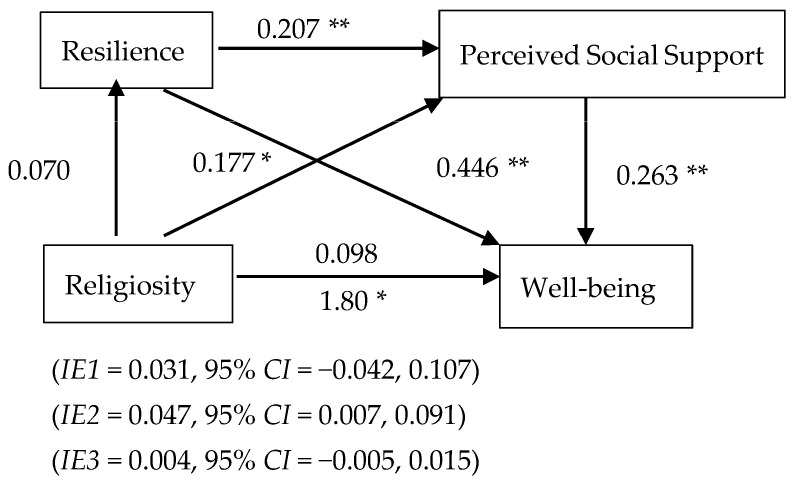
Statistical model for religiosity, resilience, perceived social support, and well-being. IE1 refers to the indirect effect of religiosity on well-being via resilience. IE2 refers to the indirect effect of religiosity on well-being via perceived social support. IE3 refers to the indirect effect of religiosity on well-being via resilience and perceived social support. Note: * *p* < 0.05, ** *p* < 0.01.

The predictor in the second mediation analysis is religiosity. The two mediators in this analysis were resilience, operationalized as scores from the Brief Resilience Scale (BRS), and social support, operationalized as perceived social support using scores from the Multidimensional Scale of Perceived Social Support (MSPSS). The covariate variable in this study was exercise behavior. The diagram is shown in Figure 2.

### 2.4. Participants

An a priori G * Power 3.1 analysis [86] with four predictors, power = 0.95, minimum effect size (f2 = 0.15), and an alpha level = 0.05, was used to calculate the recommended minimum sample size to be 129. Convenience and snowball sampling methods were used to recruit participants between November 2021 to June 2022 via the James Cook University (JCU) SONA system to manage the data collection process and disseminate credit points to eligible participants. Study links and QR codes with information about the study were shared through social media platforms (e.g., WhatsApp and Telegram groups) via the researchers’ network. Eligible students received two credit points as an incentive for taking part in the study.

This study recruited a total of 185 participants: 41 males, 136 females, 3 others (2 nonbinary, one unspecified), and 5 who did not indicate; 7 participants had bulk missing data, leaving 178 participants. The participants were between 17 to 42 years old with 10 participants withholding their age (Mage = 21.73 + 4.42). Most participants were from Singapore (N = 177), one from Australia, and seven unspecified. Participants’ affiliated religions are shown in Table 1.

### 2.5. Demographics

Participants answered a set of questions that included one set for demographics: age, gender, country of residence, and religion. Age and religion were open-ended questions. The rest of the survey was made up of the following inventories.

#### Interreligious Centrality of Religiosity Scale (CRSi-20)

CRSi-20 [87] measures the centrality and importance of religious meaning in the personality of an individual through 20 questions with five core dimensions: intellectual, ideology, public practice, private practice, and religious experience. Some example questions on each dimension are ‘How interested are you in learning more about religious topics?’, ‘To what extent do you believe in an afterlife?’, ‘How important is it to take part in religious service?’, ‘How important is personal prayer for you?’, ‘Do you experience situations in which you have the feeling that God or something divine intervenes in your life?’, respectively. For the private practice and experience dimensions, additional items were added to items 4, 5, 9, 10, and 14 for interreligious understanding.

Participants were to rate using a 5-, 6-, and 8-point Likert Scale. For items 2, 6, 7, 8, 9 (b), 12, and 13: (Not at all = 1, Not very much = 2, Moderately = 3, Quite a bit = 4, Very much so = 5). Items 1, 5 (b), 10 (b), 11, 14 (b), and 15: (Never = 1, Rarely = 2, Occasionally = 3, Often = 4, Very often = 5). Item 3 (Never = 1, Less often = 2, A few times a year = 3, One or three times a month = 4, Once a week = 5, More than once a week = 6). Items 4 (b): (Never = 1, Less often = 2, A few times a year = 3, One or three times a month = 4, Once a week = 5, More than once a week = 6, Once a day = 7, Several times a day = 8). For the 6- and 8- point Likert Scale, scorings were recoded into five levels. For the 6-point Likert Scale, except for the last two, ratings stayed the same and were recoded into a score of 5. For the 8-point Likert Scale, with (Never = 1), subsequent scoring combined two ratings (e.g., Less often or A few times a year = 2) until it reached 5. For additional items, only the higher score of both questions was included in the total calculation (e.g., in questions 4 and 4b, the one with the higher score was taken). The overall CRSi-20 score was derived by summing all subscales and dividing by 15, with a score ranging from 1.00 (not religious) to 5.00 (highly religious).

CRSi-20 has good reliability and validity and demonstrated good convergent validity among the subscales and very good total score internal consistency ranging from α = 0.92 to α = 0.96, and subscales score ranging from α = 0.71 to α = 0.93 [76,77,88].

### 2.6. Brief Resilience Scale (BRS)

BRS [46] is a 6-item scale that measures an individual’s ability to bounce back from setbacks. Items 1, 3, and 5 are positively worded items, for example, ‘I tend to bounce back quickly after hard times. Items 2, 4, and 6 are negatively worded items, for example, ‘I tend to take a long time to get over setbacks in my life’. Participants were to rate the items on a 5-point Likert Scale (Strongly Disagree = 1, Neutral = 3, Strongly Agree = 5). Negatively worded items were reverse-scored (Strongly Agree = 1, Neutral = 3, Strongly Disagree = 5). Total scores were calculated by summing all item scores and dividing the score by the total number of questions answered. Scores of BRS were continuous, with 1 being low resilience and 5 being high resilience.

BRS had good criteria and construct validity [46,89]. BRS also demonstrated good internal consistency with Cronbach values ranging from α = 0.71 to α = 0.85 in university students [89,90].

### 2.7. Multidimensional Scale of Perceived Social Support (MSPSS)

MSPSS is a 12-item scale that measures an individual’s perceived support from family, friends, and significant others [91]. Example questions for each dimension are ‘My family really tries to help me’, ‘My friends really try to help me’, and ‘There is a special person who is around when I am in need’, respectively. Participants were to rate on a 7-point Likert Scale (Very Strong Disagree = 1, Mildly Disagree = 3, Neutral = 4, Mildly Agree = 5, Very Strongly Agree = 7). The total MSPSS score could be calculated by summing all the item scores and dividing them by the total question number. Items are on a continuous scale, with 1.0 being low support and 7.0 being high support.

MSPSS demonstrated good internal consistency with Cronbach values ranging from α = 0.70 to α = 0.95 [92,93,94]. MSPSS also showed good concurrent validity [94,95,96,97] and construct validity correlating with depression and anxiety measures [98].

### 2.8. Warwick–Edinburg Mental Well-Being Scale (WEMWBS)

The WEMWBS is a 14-item scale that measured functional and emotional well-being [99]. All items are positively worded, for example, ‘I’ve been feeling relaxed’. Participants were to rate on a 5-point Likert Scale (None of the time = 1, Rarely = 2, Some of the time = 3, Often = 4, All the time = 5). Total scores were derived by summing all items. Scores of WEMWBS are continuous, ranging from 14 to 70, where a higher score reflected a higher level of mental well-being.

WEMWBS showed good internal consistency and reliability with Cronbach values ranging from α = 0.89 to α = 0.93 [100,101,102]. Test re-test reliability was also high at 0.83 [99,101]. WEMWBS also demonstrated good discriminant, construct, and content validity [101,102,103,104].

### 2.9. Paranormal Belief Questionnaire (PBQ)

PBQ examined beliefs in the supernatural such as paranormal, religion, luck, and objective morality beliefs. It was created by the corresponding author for this first use and consists of 7 items (5 choice questions and two open-ended questions). An example of a choice question can be, ‘What best describes your beliefs on religion?’ and participants can choose, ‘There is one God’ (monotheists), ‘There are multiple Gods’ (polytheists), ‘There is no God’ (atheists), ‘There might be a God, or Gods’ (agnostics), and ‘Everything is God’ (pantheists). An example of an open-ended question is, ‘Did you have any experiences with paranormal beings before? If yes, please elaborate and provide details. If no, and you believe in the existence of paranormal beings, please tell us why’. Each of the questions could be a variable on its own and used to classify participants, which, in this study, are grouped into theists and atheists. The items of PBQ can be found in the Supplementary Materials. For this study, only question one on theism was used while the rest of the questions were analyzed separately for another report.

### 2.10. Exercise Behaviors

To measure exercise behaviors, questions about the exercise behaviors of the participants (Supplementary Materials) such as, ‘How often do you exercise during the pandemic?’. Participants were to select their frequency by the time period (0 = ‘Do not exercise’, 1 = ‘Once a month’, 2 = ‘Once a week’, 3 = ‘Twice a week’, 4 = ‘Everyday’).

### 2.11. Procedure

Ethics approval was obtained from James Cook University Australia Human Research Ethics Committee (Approval Number: H8561) before beginning the online study. Participants accessed the survey through the SONA system or links and QR codes shared through different media platforms. The information sheet was presented first, followed by informed consent to which they would click ‘Yes, I understand the information and that my data will be anonymous, I wish to proceed’ and ‘Agree’, respectively, to continue. If they did not wish to take part in the study, they could close the browser or click on the ‘No, I do not wish to proceed’ or ‘Disagree’ to exit with no repercussions.

After consenting, they would proceed on to demographic questions followed by PBQ, CRSi-20, MSPSS, BRS, and WEMWBS. Participants need to click ‘→’ to proceed after each section. Upon completion of the survey, participants were thanked.

### 2.12. Mediation Analysis

According to Hayes [105], mediation occurs if (a) theism (IV) significantly predicted well-being (DV), (b) theism (IV) significantly predicted religiosity (mediator), (c) religiosity (mediator) significantly predicted well-being (DV), (d) coefficient for religious beliefs and well-being becomes nonsignificant, and (e) standardized indirect effect (IE) of religious beliefs through religiosity is nonzero. Additionally, the standardized 95% bootstrap confidence intervals (CI) of IE must exclude zero.

Hayes (2018) PROCESS macro-Model 4 with 5000 Bootstrap resamples was used to conduct this mediation hypothesis analysis. Weighted contrast codes were used on theism coded as 1 and atheism coded as 0. Theists consisted of participants who indicated that ‘Everything is God’ (Pantheists), ‘There might be a God or Gods’ (Agnostics), ‘There are multiple Gods’ (Polytheists), and ‘There is one God’ (Monotheists). Participants who indicated ‘There is no God’ were categorized as Atheists. With seven missing data being excluded from the analysis for this question in the PBQ, the total number of participants used for this analysis was N = 178. Exercise was included in this model but was not significant in both mediation analyses.

## 3. Results

### 3.1. Assumption Testing

Three outliers were detected in the boxplot diagram but were retained for analysis since they were negligible. Seven participants had empty entries and were removed, and one participant did not complete BRS5 and was thus excluded from Total BRS Score analysis. The total number of participants for the study was thus n = 178. Assumptions for normality, linearity, and homoscedasticity were also tested. From the normal P-P plots graph and scatterplot diagram, the data were shown to be normally distributed, meeting the assumption for normality. Inspection of the scatterplot showed the absence of any pattern, indicating that assumptions for linearity and homoscedasticity were also met. Lastly, the assumption test for multicollinearity showed that between theism and CRSi-20, the tolerance value was more than 0.1 with a VIF = 1.23, showing that these two predictors were not multicollinear with one another. Furthermore, between CRSi-20, BRS, MSPSS, and exercise behavior, the tolerance value was more than 0.1 and a VIF < 5, showing that these predictors were not multicollinear with each other, thus meeting the assumption for multicollinearity.

### 3.2. Hypotheses Testing—Mediation Analysis 1

#### 3.2.1. Correlation between Variables

There was no significant correlation between theism and well-being, r (178) = 0.049, *p* = 0.515. There was a moderate positive relationship found between theism and religiosity, r (178) = 0.432, *p* < 0.001, where theists expectedly had significantly higher religiosity scores than atheists. There was a very weak positive relationship between religiosity and well-being, r (178) = 0.181, *p* = 0.015. Additionally, none of them had a significant correlation with exercise. A summary of the correlations is shown in Table 2.

#### 3.2.2. Mediation Analysis

**Hypothesis** **1** **(H1).** *Theists would report higher well-being than atheists.*

After controlling for religiosity, theism did not significantly predict well-being among participants, B = −0.965 β = −0.0998, t = −0.436, *p* = 0.663. Therefore, the hypothesis was rejected. The overall total effect also showed that theism did not significantly predict well-being, B = 1.32, β = 0.137, t = 0.653, *p* = 0.515.

**Hypothesis** **2** **(H2).** *Those with higher religiosity scores would report higher well-being.*

After controlling for theism, religiosity was found to be significantly associated with the increased well-being of participants, B = 1.92, β = 0.197, t = 2.39, *p* = 0.0179. Participants with higher religiosity had better well-being scores, thus hypothesis 2 was accepted.

**Hypothesis** **3** **(H3).** *Theists would report higher well-being due to higher religiosity.*

Theists expectedly had significantly higher religiosity than the atheists, B = 1.19, β = 1.20, t = 6.35, *p* < 0.001. Regarding the indirect effect of theism on well-being, results showed that theists had higher partially standardized indirect effects on well-being due to higher religiosity, IE = 0.236, SE = 0.114, 95% CI = [0.0224, 0.468]. Since the indirect effect is more than zero, religiosity is therefore a significant mediator in the relationship between theism and well-being. The statistical diagram of this accepted hypothesis is shown in Figure 1. Additionally, the proportion mediated (PM) is 0.173.

### 3.3. Hypotheses Testing—Mediation Analysis 2

#### 3.3.1. Correlations between Variables

To investigate the relationship between the IV (religiosity), mediators (resilience and social support), DV (well-being), and the confounding variable (exercise behavior), correlations were used. There was no significant relationship between religiosity and resilience, rresilience (177) = 0.070, *p* = 0.357, but there was a small positive relationship between religiosity and perceived social support, rsocial support (178) = 0.185, *p* = 0.013. A weak positive relationship between religiosity and well-being, r (178) = 0.181 *p* = 0.015, was also found.

There was a moderate positive relationship between well-being and both mediators, rresilience (177) = 0.510, *p* < 0.001, and rsocial support (178) = 0.376, *p* < 0.001 of resilience and perceived social support, respectively. There was a weak positive relationship between resilience and perceived social support, r (177) = 0.219, *p* = 0.003, but no significant relationship between exercise behaviors and religiosity, r (178) = 0.035, *p* = 0.642, or both mediators, rresilience (177) = 0.055, *p* = 0.467, and rsocial support (178) = 0.030, *p* = 0.688 of resilience and perceived social support, respectively. Similarly, the relationship between exercise behaviors and well-being was r (178) = 0.068, *p* = 0.364.

A summary of the correlations is shown in Table 3.

#### 3.3.2. Mediation Analysis

Using the same mediation criteria as the previous mediation analysis, Hayes (2018) PROCESS macro-Model 6 with 5000 Bootstrap resamples was used to conduct this mediation analysis. Exercise behaviors were coded into an ordinal scale. Theism was excluded in this model as it was not significant in the previous analysis, and neither was exercise. Due to the exclusion of seven missing data, the total number of participants in this analysis is N = 178.

Controlling for resilience and social support, religiosity did not significantly predict well-being among participants, B = 0.959 β = 0.098, t = 1.57, *p* = 0.120, nor were there any direct effect of religiosity on well-being. In fact, the overall total effect showed that religiosity could significantly predicted well-being, B = 1.75 β = 0.180, t = 2.41, *p* = 0.017.

**Hypothesis** **4** **(H4).** *Higher religiosity scores would be associated with higher resilience and social support to result in higher well-being.*

Controlling for social support, religiosity did not significantly predict resilience, B = 0.055 β = 0.070, t = 0.923, *p* = 0.357. However, after controlling for religiosity and perceived social support, resilience was significantly positively associated to well-being, B = 5.50 β = 0.446, t = 7.06, *p* < 0.0001. The indirect effect of religiosity on well-being via resilience was not significant, IE1 = 0.031, SE = 0.037, 95% CI = [−0.042, 0.107], showing that resilience was not a significant mediator in the relationship.

Controlling for resilience, religiosity significantly predicted perceived social support, B = 0.181 β = 0.177, t = 2.42, *p* = 0.016. This indicated that participants with higher religiosity scores perceived receiving higher social support. Controlling for resilience and religiosity, perceived social support significantly increased well-being among participants, B = 2.51 β = 0.263, t = 4.10, *p* = 0.0001. The indirect effect of religiosity on well-being via perceived social support was significant, IE2 = 0.047, SE = 0.022, 95% CI = [0.007, 0.091]. Since the indirect effect was significantly greater than zero, perceived social support was a significant mediator in the relationship between religiosity and well-being.

Controlling for religiosity, resilience significantly predicted perceived social support, B = 0.268 β = 0.207, t = 2.83, *p* = 0.005. The indirect effect of religiosity on well-being via resilience and perceived social support was not significant, IE3 = 0.004, SE = 0.005, 95% CI = [−0.005, 0.015]. Additionally, the PM is 0.456. Thus, the hypothesis was rejected, and the statistical model of the hypothesis is shown as Figure 2.

## 4. Discussion

This study aimed to investigate the effects of theism and religiosity on the well-being of university students during the COVID-19 pandemic, and whether other factors such as social support and resilience mitigated the relationship. Hypothesis 1 on theism and well-being was rejected due to the similar well-being scores between theists and atheists. This was in agreement with Galen’s study [106], finding no difference between the two groups, but was contrary to previous literature that showed theism to be associated with better well-being [29]. Such differences could be due to the participant imbalance in our study of atheists (N = 27) and theists (N = 151), although another possible reason could be the differences in culture of our participants and those in the Tan, Su, Ting, Allotey, and Reidpath [29] study of university students in Australia. There may also be a need for deeper separation of theists and those affiliated with organized religions since there can be a decoupling of beliefs and adherence to religious customs and rites for people born into a family or country with a particular official religion. It should be noted that organized religions in Singapore were also heavily secularized given its identification as a secular country to maintain its religious harmony [107]. This underlying possibility was supported by earlier studies showing that secular countries had weak or nonexistent relationships between well-being and religion affiliations [108].

Participants in countries with an official religion were more inclined to turn to religion during distress than those in secular countries [80,81]. Given the emphasis on fairness and neutrality of all religions [109] in Singapore, this could have led to individuals turning to religion only during times of extreme distress and after having exhausted all other coping resources [80,81,108]. Support for this was found from unpublished qualitative analysis, where one of the open-ended questions showed that most of the participants indicated that their interest in religion did not change because they were able to keep the same routine, and that religion has no relation to the pandemic. This suggests that the participants considered the effects of the pandemic to be still within their threshold coping levels.

Hypothesis 2 was accepted given that correlation and mediation analyses demonstrated that higher religiosity indicated better well-being. The findings agreed with previous literature showing positive associations between religiosity and well-being [30,110,111]. Religiosity, along with its implied observance of rites and customs, created a sense of belonging and purpose in individuals, promoting positive effect and well-being [112]. This could have helped our participants maintain their religiosity and well-being during the pandemic. 

Hypothesis 3 was also accepted in which mediation analysis showed that people’s religious beliefs would report higher well-being due to higher religiosity.

As one of the first few studies in our knowledge to incorporate theism, religiosity, and well-being in one model, our findings agreed with previous literature on the direct effects of each pathway [11,29,30], except for the impact of religious beliefs on well-being. Religiosity was thus a mediating factor between the two and offered cognitive and emotional resources to manage uncertainties and overcome difficulties [113]. Theism on its own did not have a direct impact, but it was rather through religiosity. A strong believer would more often have high religiosity in developing social connections formed with the congregation [114] of fellow believers for more support in times of need. While most previous studies focused on religious attendance contributing to better well-being [30,110,111], the intrinsic factor of religiosity could also be part of the relationship. Intrinsic religiosity, defined as the ‘try to consistently live the religion they believe’ [115] could also explain the relationship that was also supported by Steffen et al. [116], in which individuals with higher intrinsic religiosity had better well-being by integrating their religion into their daily lives. This integration buffered anxiety and fear of death [117], placing less stress on an individual, thus predicting greater well-being. Considering the rejection of hypothesis 1 on theism and well-being, our findings here strongly supported the social support in religion to be the main contributor of positive effects rather than their beliefs alone.

Hypothesis 4, where higher resilience and higher perceived social support together did not mediate religiosity and well-being, was thus rejected. As one of the first few studies to incorporate resilience and perceived social support on religiosity and well-being in a sequential mediation model while controlling exercise behaviors as a covariate, our results on direct pathways were consistent with the literature [73,83], but not for the association between religiosity and resilience [75]. Nonetheless, owing to the lack of association between religiosity and resilience or a role for exercise, the model and hypothesis were rejected.

On its own, perceived social support was a mediator between religiosity and well-being, and this was supported by the various direct associations between the variables [83]. Li, Luo, Mu, Li, Ye, Zheng, Xu, Ding, Ling, Zhou, and Chen [36] found social support to mediate between religiosity and life satisfaction, possibly due to the social factor of both perceived social support and religiosity in a possible socially based religiosity that could account for better well-being.

Despite resilience not being a mediator in the relationship between religiosity and well-being, our study showed resilience to have a positive relationship with well-being.

Relevant to well-being during times of crises, public health interventions could focus on the social support of the population given that we found social support to mediate between religiosity and well-being. While some social support can comesfrom the practice of religious rites and customs in the high religiosity group, effort could perhaps be made for better well-being of those not belonging to any organized religion, especially during large-scale crises.

### Limitations and Future Work

The inconsistency of our findings on resilience with previous literature may be due to the different measures of resilience used. Most studies used either the Connor–Davidson Resilience Scale or the Wagnild and Young Resilience Scale as measures of resilience [63,73,74]. However, Schwalm et al. [118] found that both measures had religious components different from the Brief Resilience Scale, which did not take religious components into account. For example, ‘Sometimes fate or God can help’ or ‘my life has a meaning’ overlapped religiosity and spirituality [118], making it difficult to separate the effects. In addition, we intentionally separated theism from religiosity given that the two may not be coupled where people may adhere to religious norms out of family/national or even social obligations as opposed to actual personal belief. As mentioned in the introduction, there could be a distinction between staunch and social-orientated theists, and there could also be impact between the different theists. For example, pantheists who believe that everything is or has some god element may be more inclined to accept situations as part and parcel of things, whereas polytheists may choose to pray to a particular entity to act on the situation believed to be caused by another entity. Such differences would naturally lead to different coping mechanisms and responses to crises, thereby impacting resilience. It should also be noted that even within the same type of theism, there are many parameters such as faith, religious experience, religious knowledge at play that could be major varying factors, leaving much to investigate for future studies.

## 5. Conclusions

We found some protective effects from theistic beliefs and religiosity on well-being through resilience and perceived social support of university students during the COVID-19 pandemic in Singapore. Religiosity mitigated the relationship between theism and well-being. Despite not showing significant results with resilience and perceived social support as sequential mediators in this study, these parameters improved well-being during the COVID-19 pandemic, with relevance for future intervention strategies and preparation for inevitable global crises to come.

## Figures and Tables

**Table 1:** The religious demographics of the participants.

Religion	Number of Participants
Christian	39 (21.1%)
Buddhist	38 (20.5%)
Hindu	11 (5.9%)
Catholic	9 (4.9%)
Muslim	8 (4.3%)
Other Religions	14 (7.6%)
Agnostic	8 (4.3%)
No Religion	24 (13.0%)
Freethinkers	16 (8.6%)
Atheist	3 (1.6%)
Unspecified	15 (8.1%)

**Table 2:** Correlations, means, and standard deviations between Mediation 1 variables.

Variables	*M*(*SD*)	1	2	3	4
1. Theism	-	-			
2. Total Score on Interreligious Centrality of Religiosity Scale	2.77(0.994)	0.432 **	-		
3. Total Score on Warwick–Edinburg Mental Well-being Scale	45.0(9.67)	0.049	0.181 *	-	
4. Exercise	2.04(1.51)	0.084	0.035	0.068	-

Note: * *p* < 0.05, ** *p* < 0.01.

**Table 3:** Correlations, means, and standard deviations between Mediation 2 variables.

Variables	*M*(*SD*)	1	2	3	4	5
1. Total Score on Interreligious Centrality of Religiosity Scale	2.77 (0.994)	-				
2. Total Score on the Brief Resilience Scale	3.02 (0.785)	0.070	-			
3. Total Score on Multidimensional Scale of Perceived Social Support	5.12 (1.02)	0.185 *	0.219 **	-		
4. Total Score on Warwick–Edinburg Mental Well-being Scale	45.0 (9.67)	0.181 *	0.510 **	0.376 **	-	
5. Exercise	2.04 (1.51)	0.035	0.055	0.030	0.068	-

Note. * *p* < 0.05, ** *p* < 0.01.

## Data Availability

Data available from the corresponding author upon reasonable request.

## Supplementary Materials

The following supporting information can be downloaded at: https://www.mdpi.com/article/10.3390/ijerph20043620/s1, S1: Demographic questions; S2: Paranormal Belief Questionnaire; S3: Exercise behaviors and open-ended questions of study.
