# Supplementary material for: Religiosity, Theism, Perceived Social Support, Resilience, and Well-Being of University Undergraduate Students in Singapore during the COVID-19 Pandemic
Source: Int J Environ Res Public Health. 2023 Feb 17;20(4):3620. doi: 10.3390/ijerph20043620 (PMC9959174; doi:10.3390/ijerph20043620)
Supplement: Supplementary file 1 [file ijerph-20-03620-s001.zip › ijerph-2143679-supplementary.pdf]

International Journal of
Environmental Research and Public Health

MDPI

Supplementary Material

S1. Demographic Questions of Study

Q1
Section 1: Demographics
Please indicate your gender:

- ○ Male
- ○ Female
- ○ Others: ____________________

Q2 Please indicate your religion:

____

Q3 Indicate country of campus:

- ○ Singapore
- ○ Australia

Q4 Please indicate your age:

____

S2. Paranormal Belief Questionnaire

Section 2.1: Religion Parameters

Q1) What best describes your beliefs on religion?

- ○ There is one God
- ○ There are multiple Gods
- ○ There is no God
- ○ There might be a God, or Gods
- ○ Everything Is God

Q2) Do you believe in paranormal beings (i.e. spirits, demons, ghosts)?

- ○ I do not believe in any of them
- ○ Spirits of the dead (e.g. ghost, Pontianak, ancestors watching over, etc.)
- ○ Spirits/beings connected to nature or objects (e.g., Mother Nature, poltergeists, etc.)
- ○ Folklore beings (e.g., fairies, vampires, werewolves, zombies, dragons, the Kraken, Moon Rabbit, etc.)
- ○ Cryptid creatures (e.g., Bigfoot, Yeti, Loch Ness Monster, Phantom Cats, etc.) (5)
- ○ Angels and demons
- ○ Others

Q3) Did you have any experience with paranormal beings before? If yes, please elaborate and provide details. If no, and you believe in the existence of paranormal beings, please tell us why. Otherwise, please indicate "NA":

____

Q4) What best describes your beliefs on Extra-terrestrials/Aliens (i.e. sentient beings from another planet)?

- They do not exist.
- They exist but are more primitive than humans (not technologically advanced).
- They exist, but do not contact us.
- They exist; they secretly maintain contact with some humans, from outside Earth.
- They exists; they made contact and now live among us.
- They exist; they made contact and humans serve(d) them as Gods.
- Others

Q5) Have you had an encounter with an Extra-terrestrial (ET)? If yes, please elaborate and provide details. If no, and you believe in the existence of ETs, please tell us why. Otherwise, please indicate "NA"

____

Q6) Do you have beliefs or practices that are considered superstitious, or related to luck/fortune? (includes talismans, charms, horoscopes/astrology, asking advice from the dead for luck, etc)

- Yes
- No, I do not believe in luck.
- No, I do not have such practices, but I believe in luck.

Q7) Do you believe in objective morality? A belief that every action is not up for interpretation and can never be debated on whether it is right or wrong. Some might think it as commandments from God, while others think it as the universe having some objective rules we may follow.

- Yes
- No
- Not sure

S3. Exercise Behaviours and Open-Ended Questions of Study

Section 6: Exercise behaviour during Covid-19 Lockdown

1) Do you usually exercise regularly?

- ○ Yes
- ○ No

2) Is there a change in frequency of your indoor exercise (i.e. gym, home exercise, etc) regime because of the pandemic?

- ○ Increase
- ○ Decrease
- ○ No change

3) How often do you exercise during the pandemic?

- ○ Once a month
- ○ Once a week
- ○ Twice a week
- ○ Everyday
- ○ Don't exercise

Section 7: Open-ended

If you are willing, you may state any extreme or severe situations arising from the pandemic may have influenced your well-being during the COVID19 pandemic, otherwise, please put NA.

____
